# Gallbladder Adenomyomatosis Mimicking Carcinoma: A Diagnostic Dilemma

**DOI:** 10.1200/JGO.2016.005249

**Published:** 2016-06-15

**Authors:** Abhishek Mahajan, Smiti Sripathi

**Affiliations:** **Abhishek Mahajan**, Tata Memorial Centre, Mumbai; and **Smiti Sripathi**, Kasturba Medical College, Manipal, India.

## CASE REPORT

A 69-year-old man presented with right hypochondriac pain accompanied by postprandial nausea and vomiting for 2 months. Additional symptoms included fever and fatigue for 4 days. There was associated history of weight loss of approximately 6 kg over the past few months. Vital signs were normal, and he was febrile (38.3°C). His skin and sclerae were mildly icteric. He had no signs of liver failure. His abdomen was soft, with tenderness noted in the right hypochondriac region (Murphy sign positive); no tenderness was noted elsewhere, and there was no evidence of ascites. The tip of the gallbladder was palpable in the right 9th costal margin. Laboratory evaluation revealed mild conjugated hyperbilirubinemia (total bilirubin level, 2.1 mg/dL; direct bilirubin level, 1.1 mg/dL), with normal levels of transaminases and alkaline phosphatase. Mild hypoalbuminemia was present. Other investigations were unremarkable. On the basis of the clinical and laboratory findings, the patient was evaluated for obstructive jaundice, and ultrasonography (US) was performed.

## IMAGING FINDINGS

**Fig 1:**
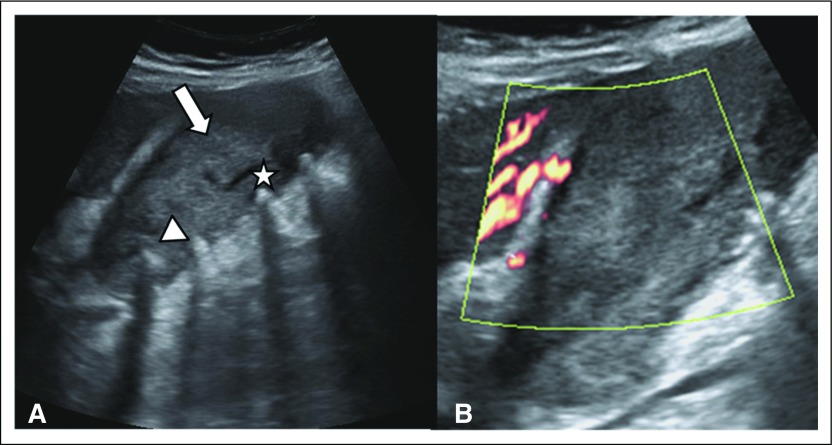
– Ultrasonographic images of gallbladder in a 69-year-old man with right hypochondriac pain. (A) Longitudinal ultrasonographic image shows diffusely thickened gallbladder wall (arrow) with associated multiple intramural calculi (arrowhead) and echogenic intramural foci with comet-tail reverberation artifacts, indicative of cholesterol crystals within Rokitansky-Aschoff sinuses (star). (B) No obvious color uptake on power Doppler.

US revealed an abnormally thick gallbladder wall measuring 3 cm in thickness. The wall thickening gave the appearance of an endophytic mass causing near-total obliteration of the lumen. Multiple intraluminal and intramural calculi were seen (Fig 1A). Few echogenic intramural foci were also seen. No color uptake was noted on power Doppler evaluation (Fig 1B). Sonographic Murphy sign was absent, and there was no pericholecystic fluid collection. There was no extra or intrahepatic biliary system dilatation. Gallbladder malignancy/adenomyomatosis with cholelithiasis was considered as the probable diagnosis; in view of absence of acute inflammatory features, cholecystitis was considered unlikely. No other sonographic abnormality was identified.

## PATHOLOGIC EVALUATION

Interval cholecystectomy was performed 4 weeks after initial presentation. At laparotomy, diffusely enlarged gallbladder was seen, which was firm and showed multiple adhesions.

**Fig 2:**
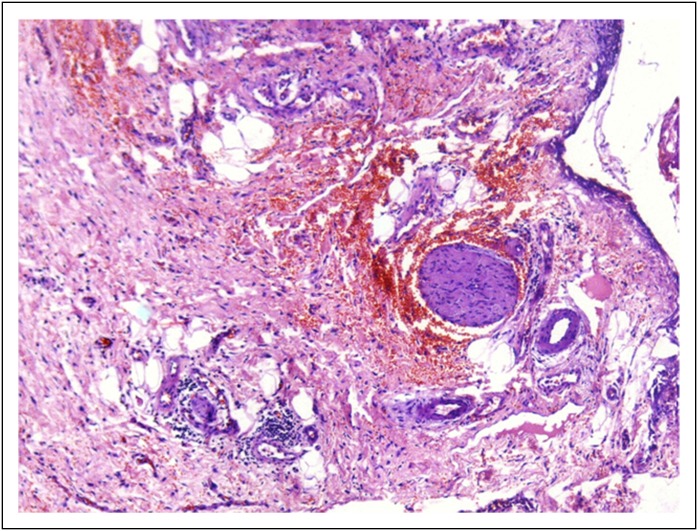
– Photomicrograph (original magnification, ×20; hematoxylin and eosin stain) showed ulcerations and edematous gallbladder mucosa and myoepithelial hyperplasia with abundant granulation tissue composed of myofibroblasts. Inflammatory cells, such as neutrophils, plasma cells, and macrophages, were also present with congested vessels.

A gross cholecystectomy specimen showed diffusely enlarged gallbladder weighing 63 g and measuring 7.5 × 5.5 × 2.5 cm. On cut sections, the gallbladder was firm, with grayish thickened wall measuring up to 2.5 cm and velvety granular mucosa containing numerous multifaceted intraluminal calculi. The gallbladder wall showed areas of hemorrhages and contained multiple pockets, many of which also contained calculi. Microsections showed ulcerations and edematous gallbladder mucosa and myoepithelial hyperplasia with abundant granulation tissue composed of myofibroblasts. Inflammatory cells, such as neutrophils, plasma cells, and macrophages, were also present, with congested vessels (Fig 2). The final diagnosis of adenomyomatosis of the gallbladder was confirmed by the characteristic histopathologic appearance of muscular and epithelial hyperplasia contributing to mural thickening, with epithelial invaginations forming the pathognomonic intramural diverticula known as Rokitansky-Aschoff sinuses (Fig 2). No evidence of malignancy was found.

## DISCUSSION

Adenomyomatosis is not an uncommon mimic of the gallbladder carcinoma, identified in 1% to 8.5% of cholecystectomy specimens.^1^ Table 1 summaries the clinicoradiopathological features of gallbladder adenomyomatosis. It is most often an incidental finding, has no intrinsic malignant potential, and usually requires no specific treatment.^2,3^ Varied names have been applied to this lesion in the literature, including adenomyomatosis, adenomyoma, diverticular disease, intramural diverticulosis, cholecystitis cystica, and cholecystitis glandularis proliferans. It is more commonly seen in women than in men, the majority presenting with complaints of chronic right upper quadrant pain.^2^ Frequent coexistence with cholelithiasis is seen; however, no causative relationship has been proved.^4^ Adenomyomatosis occasionally produces abdominal pain, and in some cases, cholecystectomy may be indicated for relief of symptoms.^4^

**Table 1:**
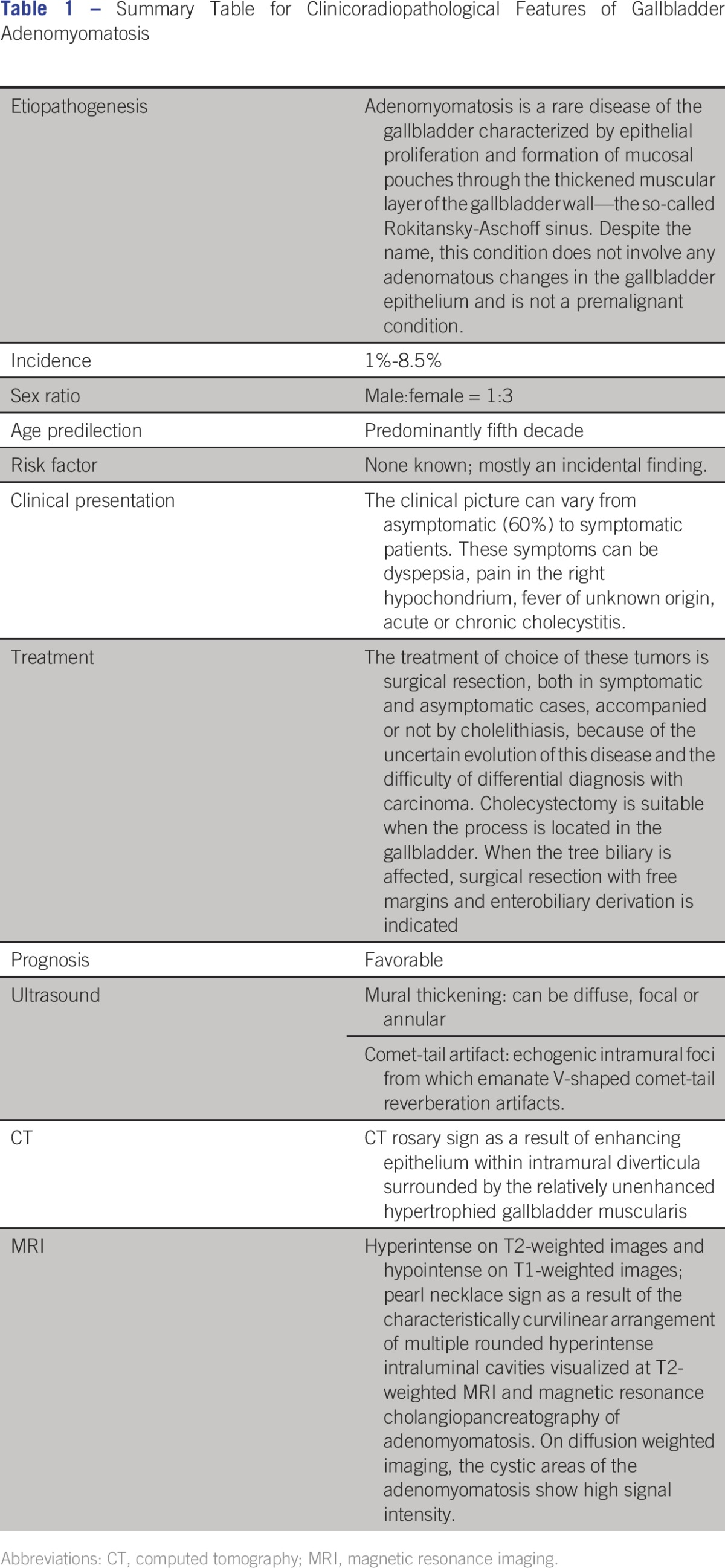
Summary Table for Clinicoradiopathological Features of Gallbladder Adenomyomatosis

Dysplasia, carcinoma in situ, and invasive carcinomas may arise from the epithelium of adenomyomatous hyperplasia. However, most authors believe that the cause for development of carcinoma in adenomyomatous hyperplasia is the presence of stones, chronic inflammation, and metaplastic changes rather than adenomyomatous hyperplasia itself. Thus, adenomyomatous hyperplasia is not considered a premalignant lesion.^2-4^

US is considered as the baseline imaging modality to investigate the hepatobiliary system, and adenomyomatosis of the gallbladder is a not-infrequent pathology, seen in approximately 2% of patients.^1,5^ Evaluation of gallbladder wall thickness plays an important role in the sonographic study of the biliary system. The gallbladder wall is no more than 2 mm thick in 97% of healthy subjects, if the short axis of the gallbladder is at least 2 cm in diameter.^5^ Diffuse gallbladder wall thickening has a differential diagnosis that includes the more common inflammatory and noninflammatory causes of wall thickening. The various causes include hepatitis, artifacts induced by the presence of pericholecystic fluid, hypoalbuminemia, portal hypertension, right-sided heart failure, incomplete gallbladder distention, cirrhosis, hepatitis, and cholecystitis. In addition, focal thickening occurs in gallbladder carcinoma and adenomyomatosis.^5,6^ However, gallbladder malignancy should be suspected when there are features of a focal mass with locoregional lymphadenopathy, metastases, and features of biliary obstruction at the level of the porta hepatis.^5^

The pathognomonic pathologic features of the adenomyomatosis are thickened gallbladder wall and intramural diverticula containing bile, with cholesterol crystals, sludge, or calculi that show a distinct correlation with the modality imaging findings.^7^ Longstanding Rokitansky-Aschoff sinuses of adenomyomatous hyperplasia result in calcification of the intramural sludge, cholesterol, or stones. Rarely, abdominal radiograph may show them as nondependent calcific opacities in the right upper quadrant.^7^

On ultrasonography, gallbladder involvement by adenomyomatous hyperplasia is variable in location and extent, which varies from focal to diffuse gallbladder wall thickening.^7^ Luminal narrowing may be seen in the diffuse and segmental variants that may produce a characteristic hourglass configuration.

The diagnostic sonographic hallmark on ultrasonography is the presence of the anechoic or echogenic intramural diverticula. Anechoic diverticula contain bile, whereas those that contain sludge, cholesterol, or intramural calculi are seen as echogenic foci.^4,7^ Comet-tail/V-shaped reverberation artifact is due to echogenic sound reverberation emanating from the small echogenic foci in the gallbladder wall into the central anechoic lumen. Similar appearance may be caused by intraluminal or intramural air, as seen in emphysematous cholecystitis; however, the mobile nature of the air with dirty echogenic shadowing on ultrasonography helps in differentiating them. In equivocal cases, computed tomography (CT) plays an important role.

CT findings of adenomyomatous hyperplasia usually correlate to the sonographic findings. All three pathologic forms (localized, segmental, and diffuse) are apparent on CT scans.^4,8^ Intramural cystic spaces and calculi demonstration on CT scan is not possible, but they are easily picked up sonographically because of reverberation artifact. This makes ultrasonography a primary diagnostic imaging modality for the diagnosis of adenomyomatous hyperplasia. The Rokitansky-Aschoff sinuses that are large enough may be seen on CT scan, giving a characteristic appearance, the rosary sign, which is due to the enhancing epithelium within intramural diverticula surrounded by the relatively unenhanced hypertrophied gallbladder muscularis.^9^

Magnetic resonance imaging (MRI) plays an important role in distinguishing adenomyomatous hyperplasia from malignant lesions; hyperintense intraluminal cavities visualized on T2-weighted MRI are suggestive of Rokitansky-Aschoff sinuses and have been reported to be useful.^10^ When arranged in a curvilinear pattern, they give an appearance of pearl necklace sign.^11^ The intramural calculi are seen as signal void because of the mineral content. The pearl necklace sign is specific for adenomyomatosis of the gallbladder and is not seen in gallbladder carcinoma.^11^ Differentiation of echogenic intramural foci from abnormal enhancement requires a multiphasic MRI protocol with intravenous contrast material. Diffusion weighted imaging has been found useful in differentiating gallbladder adenomyomatosis from gallbladder carcinoma and significantly improves the diagnostic accuracy.^11a^

In our case, US revealed an abnormally thick gallbladder wall measuring 3 cm in thickness. The wall thickening gave an appearance of endophytic mass causing near-total luminal obliteration. Multiple intramural and intraluminal calculi were seen with few echogenic intramural foci, which did not show the classic comet-tail reverberation artifacts. The cause for the diagnostic dilemma was the absence of the comet-tail/V-shaped reverberation artifact, which is the diagnostic sonographic hallmark of adenomyomatosis. Its absence can be explained on the basis of the absence of central anechoic gallbladder lumen because of gross dense fibrous wall thickening causing near-total luminal obliteration.^12^

Although the imaging features of adenomyomatosis can be distinctive enough to allow confident diagnosis, findings such as gallbladder wall thickening and enhancement are somewhat nonspecific. The differential diagnosis includes adenomatous, hyperplastic, and cholesterol polyposis, papillomatosis, adenoma, portal hypertension, total parenteral nutrition, hypoalbuminemia, congestive heart failure, and cystadenoma, as well as malignancies such as gallbladder adenocarcinoma, carcinoid tumor, and metastatic melanoma of the gallbladder (Table 2).

**Table 2:**
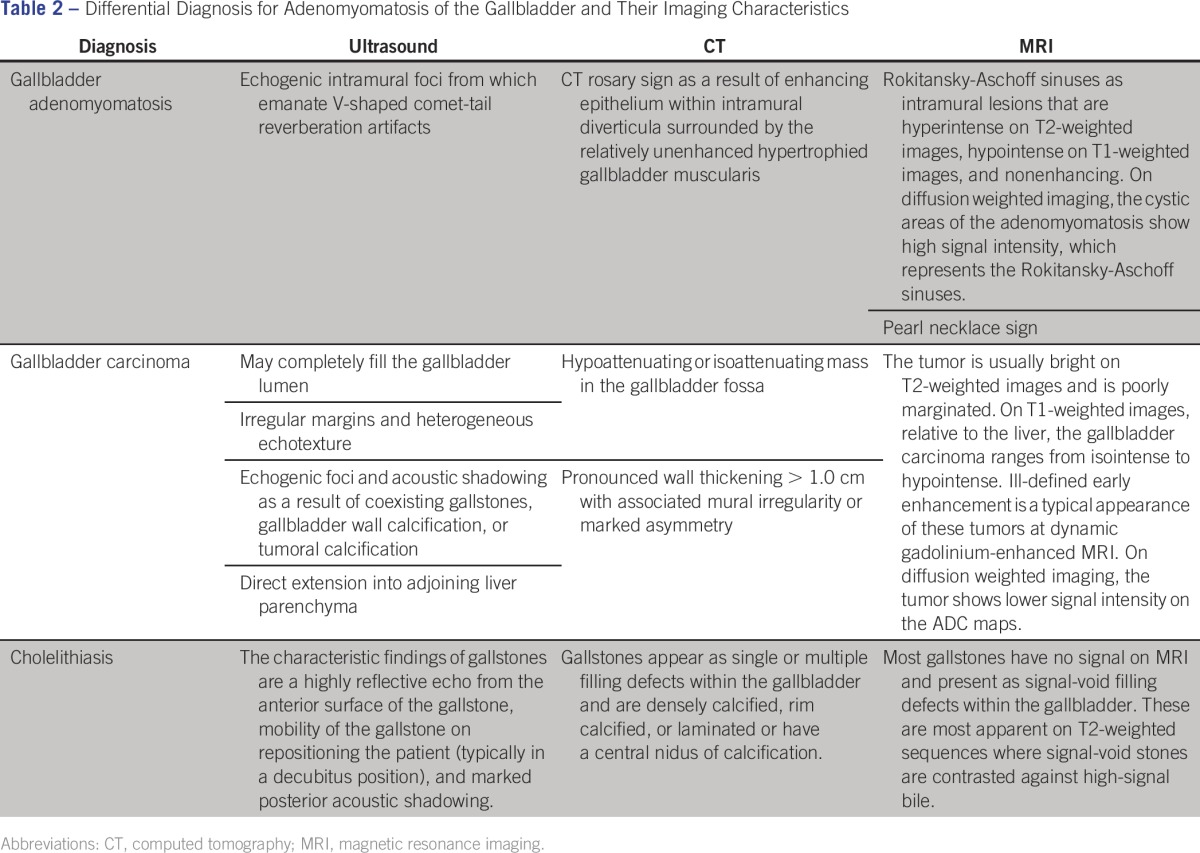
Differential Diagnosis for Adenomyomatosis of the Gallbladder and Their Imaging Characteristics

Metabolic characterization with [^18^F]fluorodeoxyglucose (FDG) positron emission tomography may be a useful adjunct in problematic cases.^13^ Adenomyomatosis does not show any FDG uptake on positron emission tomography study, which makes it a problem-solving tool in differentiating it from malignant gallbladder wall thickening, which shows focal or diffuse FDG uptake.^13^ Though the FDG uptake within these lesions represents the metabolic activity of the underlying pathological tissue (cancerous cells), false positive results may also be seen in cases with associated active inflammatory process such as cholecystitis.^14^

In conclusion, great difficulty arises in differentiating the diffuse variant of adenomyomatous hyperplasia that simulates a gallbladder malignancy, especially in the absence of the diagnostic hallmark comet-tail/V-shaped reverberation artifact, which may be absent in cases of total luminal obliteration by the pathology. Surgical resection may be indicated in symptomatic cases and when nonspecific findings present a diagnostic dilemma.
